# Clinical and Microbiological Periodontal Biofilm Evaluation of Patients with Type I Diabetes

**DOI:** 10.3390/jcm13226724

**Published:** 2024-11-08

**Authors:** Mihaela Maris, Maria-Alexandra Martu, Marius Maris, Cristian Martu, Diana Maria Anton, Mariana Pacurar, Kamel Earar

**Affiliations:** 1 Faculty of Dental Medicine, University of Medicine and Pharmacy “Dunărea de Jos”, 800201 Galati, Romania; mihaela@drmaris.ro (M.M.); erar_dr.kamel@yahoo.com (K.E.); 2 Faculty of Dental Medicine, “Grigore T. Popa” University of Medicine and Pharmacy, 700115 Iasi, Romania; 3 Faculty of Dental Medicine, University “Titu Maiorescu”, 22 Dâmbovnicului Tineretului Street, 040441 Bucharest, Romania; marius@drmaris.ro; 4 Faculty of Medicine, ENT Clinic Department, “Grigore T. Popa” University of Medicine and Pharmacy, Universitatii Street 16, 700115 Iasi, Romania; martu.cristian@umfiasi.ro; 5 Hospital “Sf. Apostol Andrei”, 800578 Galati, Romania; diana_maria_a@yahoo.com; 6 Faculty of Dental Medicine, George Emil Palade University of Medicine, Pharmacy, Science and Technology, 38 Gheorghe Marinescu Street, 540142 Targu Mures, Romania; mariana.pacurar@umfst.ro

**Keywords:** type I diabetes, periodontitis, bacteria, glycemia, glycated hemoglobin, periodontal pocket

## Abstract

**Background/Objectives:** The purpose of this study was to assess the microbial composition and density of subgingival plaque samples for periodontal pathogens while correlating the values with glycemic control levels via glycated hemoglobin (HbA1c), a type of hemoglobin that has chemically linked glucose, in type I diabetes individuals who will undergo complex oral rehabilitation through orthodontic treatment and implant surgery. **Methods:** A cohort of 42 adults with type I diabetes were included in this study. The subjects sustained a comprehensive periodontal clinical examination as well as microbiological assessments of their subgingival plaque samples through quantitative real-time PCR. The samples were collected from the two deepest pockets of each subject. **Results:** The highest number of periodontopathogenic bacteria was observed in the pockets of 5–7 mm. *T. forsythia* showed the highest prevalence (20.48%), with decreasing numbers as follows: *T. denticola* (13.31%), *P. gingivalis* (11.26%), *A. actinomycetemcomitans* (7%), and *P. intermedia* (4.9%). *T. denticola* and *T. forsythia* were significantly more commonly observed in individuals with elevated HbA1c serum levels. No correlation was observed between *P. gingivalis*, *A. actinomycetemcomitans*, *P. intermedia* presence, and the HbA1c value. **Conclusions:** Periodontopathogenic agents’ presence in subgingival biofilm samples varied in accordance with the pocket probing depth and metabolic control of the diabetic individuals. In our study, the appearance of these periodontopathogenic agents was linked to lowered metabolic control in patients with type I diabetes mellitus.

## 1. Introduction

Periodontal diseases (gingivitis and periodontitis) are inflammatory biofilm-determined diseases that affect the tooth-supporting tissues [1,2]. Recent research detailing periodontal tissues, oral cavity, immune system, bacteria, and other microorganisms and host responses have shown that the etiopathogenesis of periodontal disease is transmuted from an exclusively bacterial origin concept to a more holistic understanding, with a true cause–effect relationship of a multifactorial nature in which genetic, exogenous, and lifestyle factors, in general, can significantly affect oral health. While a definitive causal link between periodontitis and systemic diseases has yet to be firmly established, research suggests that periodontopathogens and their resulting immune-inflammatory responses are independently linked to the development of several systemic pathologies, including atherosclerotic cardiovascular diseases, diabetes mellitus, Alzheimer’s and other neurodegenerative diseases, rheumatoid arthritis, osteoporosis, pulmonary diseases, COVID-19, cancer, and chronic kidney disease, to name a few [3,4,5,6,7,8].

Diabetes mellitus is a major global chronic disease that has reached pandemic proportions and is acknowledged as a major contributor to early morbidity and mortality worldwide. According to the 10th edition of the IDF (International Diabetes Federation) Diabetes Atlas, the global incidence of diabetes has risen to over 1 in 10 adults in 2021, with expectations for this number to grow swiftly in the future [9,10]. Additionally, diabetic patients have an increased risk of cancers such as that of the breast, liver, pancreas, colon, and urinary tract [11,12,13,14].

Until now, any distinct phenotypic traits specific to periodontitis in diabetic patients have not been observed. Therefore, diabetes-associated periodontitis is not categorized as a separate disease. However, diabetes is a significant modifier of periodontitis progression and is considered in the clinical grading system for its diagnosis [15,16,17,18]. More research has been made on the interaction between type 2 diabetes and periodontitis, and studies show that individuals with type 2 diabetes have a heightened risk of periodontal disease due to their weakened defenses against bacterial plaque (biofilm) [19,20]. Although these issues are not unique to diabetics, a higher prevalence of oral pathologies is observed in this group, which can negatively impact their quality of life through psychological, social, and functional dimensions [21,22]. The investigation of the pathophysiological aspects of the interactions between type I diabetes and periodontitis have not been analyzed properly yet, and neither have the treatment responses in this specific group of patients [23,24,25].

The purpose of this study was to assess the microbial composition and density of subgingival plaque samples for periodontal pathogens while correlating the values with glycemic control levels via glycated hemoglobin (HbA1c) in type I diabetes individuals who will undergo complex oral rehabilitation through orthodontic treatment and implant surgery.

## 2. Materials and Methods

### 2.1. Study Design

This study had a cross-sectional design and was conducted on a cohort of 42 adult individuals diagnosed with type I diabetes who will undergo complex oral rehabilitation through orthodontic and implant treatment, this study being part of a larger more complex study on the influence of periodontal status on patients with type I diabetes. This study was held at the “Grigore T. Popa” University of Medicine and Pharmacy, Iași, from November 2020 to May 2021.

This study adhered to the ethical standards set by the Declaration of Helsinki and was approved by the Ethics Committee of “Grigore T. Popa” University of Medicine and Pharmacy, Iași, on 30 July 2020. The patients were informed about the purpose of the research, and all of them provided a signed informed consent for participation.

### 2.2. Patient Selection

The inclusion criteria were type 1 diabetes patients aged 18–70 years, permanent residency in the study area, and a diagnosis for diabetes at least 1 year previously.

Patients were excluded if they had systemic diseases not related to diabetes, a history of cancer, were pregnant, were breastfeeding, were menopausal, were smokers, had undergone treatment for periodontal disease in the last 6 months, had received antibiotic treatment in the last 3 months, used medication that influences periodontal status or that can cause gingival enlargement (such as channel blockers), used antidepressants, or had fewer than 20 remaining teeth (Table 1).

### 2.3. Collection of Clinical Data

The subjects sustained a comprehensive clinical examination of their periodontal tissues, which included the following: clinical attachment loss (CAL) direct method; periodontal probing depth (PD), and bleeding on probing index (BOP). Clinical attachment loss and probing depth were measured at six sites per tooth: distobuccal, centrobuccal, mesiobuccal, mesiolingual, centrolingual, and distolingual. A CP-12 manual periodontal probe (Hu-Friedy Mfg. Co., LLC, Chicago, IL, USA) was used for periodontal clinical examination. All the data gathered from the periodontal measurements were incorporated in the patient’s individual record.

### 2.4. Microbiological Analysis

The periodontal pathogens were assessed in the subgingival plaque samples collected from the two deepest pockets of each subject with paper points. In each periodontal pocket, a sterile paper point was inserted, left in place for 30 s, and subsequently transferred to a sterile microtube (1.5 mL) containing 1 mL of RNAlater^®^ Stabilization Solution (ThermoFisher Scientific, Waltham, MA, USA).

### 2.5. DNA Extraction

DNA extraction was performed using the PureLink^®^ Genomic DNA Kits (ThermoFisher Scientific, USA) with some alterations. The steps for DNA extraction, according to the manufacturer’s guide, included lysate preparation. The materials needed for lysate preparation were 96–100% ethanol, a sample for DNA isolation in the quantity specified by the kit manufacturer, Phosphate-Buffered Saline (PBS) solution, sterile DNase-free microcentrifuge tubes, a water bath, a microcentrifuge, and solutions provided in the working kit—PureLink^®^ Genomic Digestion Buffer, PureLink^®^ Genomic Lysis/Binding Buffer, RNase A (20 mg/mL), Proteinase K (20 mg/mL).

To obtain the lysate from the blood samples, we followed the protocol recommended by the kit manufacturer. Following this step, we proceeded to DNA binding. The necessary materials for DNA binding included the following: the previously obtained lysate, sterile microcentrifuge tubes (DNase-free) of 1.5 mL for elution, a microcentrifuge, sterile water with a pH of 7.0–8.5, and materials from the working kit—Genomic Elution Buffer, Genomic Wash Buffers 1 and 2, Collection Tubes, Spin Columns in Collection Tubes (PureLink^®^). Following the kit manufacturer’s recommendations for this stage ensured optimal results. Before beginning the procedure, 96–100% ethanol was added to the PureLink^®^ Genomic Wash Buffer 1 and PureLink^®^ Genomic Wash Buffer 2 solutions as instructed, and these containers were stored at room temperature.

After following the recommended steps for DNA binding, we proceeded to DNA washing and elution. To obtain a higher quantity of DNA, we executed a second elution step: centrifuging the column at room temperature for 1.5 min at maximum speed. The resulting tube contained purified DNA. The column was removed and discarded.

For DNA storage, part of the purified DNA was stored at −20 °C for later use, while another part was stored at 4 °C for immediate further steps.

### 2.6. DNA Quantification

The purity and quantification of the isolated DNA were assessed via spectrophotometry with a NanoPhotometer^®^ (Implen Gmbh, Munich, Germany). Quantification was determined at an optical density (OD) of 260 nm, and the OD260 nm/OD280 nm ratio indicated DNA purity. Generally, OD260 nm/OD280 nm ratios of 1.7–2.0 signified pure DNA [26].

For the quantification of periodontal pathogens, the primer and probe sequences used for quantifying the studied periodontal pathogens by qPCR are shown in Table 2. The primers addressed highly conserved regions of the waaA (kdtA) and waaG genes. Both of the genes were present as single copies in the genomes of the species listed, simplifying the real quantification of the microorganisms. The result of the qPCR depicted the number of amplicons, and in this case, since the amplicon depicted a fragment of a single-copy gene, it directly translated into the genome copies number, equivalent to the number of microorganisms.

For constructing the standard curve and as a positive control, a recombinant plasmid containing the sequence of DNA of interest flanked by sequences complementary to the primers for attachment was used. This synthetic plasmid was purchased from Primer Design, UK. Decimal dilutions of the positive control from 10 to 107 copies per reaction were made. BioPure water was utilized as a negative control to substitute the isolated DNA from the sample for analysis.

The quantification of periodontal pathogens was performed via quantitative real-time PCR using the TaqMan method on the qPCR platform Mx3005P (Stratagene, San Diego, CA, USA). The qPCR reactions were conducted in a total volume of 25 µL, including 2 µL of isolated DNA from the sample, 12.5 µL of GoTaq^®^ qPCR Master Mix, 0.4 µL ROX, and optimized volumes of primers, probe, and BioPure water to establish efficient concentrations.

For *A. actinomycetemcomitans*, the primer and probe concentrations were 100 nM and 200 nM, respectively, 300 nM and 200 nM for *P. gingivalis*, 100 nM and 100 nM for *P. intermedia*, 300 nM and 100 nM for *T. denticola*, and 100 nM and 100 nM for *T. forsythia*. Amplification was realized using the following thermal program: the initial denaturation at 95 °C for 10 min, followed by 40 cycles of 95 °C for 30 s and 60 °C for 1 min.

Glycated hemoglobin A1c (HbA1c) was determined for each subject, using the immunoturbidimetric method.

### 2.7. Statistical Analysis

Data were recorded and statistically analyzed. Pearson’s correlation coefficient was used to test the relationship between the probing depth, the prevalence of periodontal pathogens, and the glycemic control.

## 3. Results

The present study was conducted on a number of 42 individuals with type I diabetes mellitus of which 18 were male patients (42.86%) and 24 were females (57.14%). The average age of the individuals analyzed in this study was 46.89 ± 1.14 years (Table 3).

**Figure 1:**
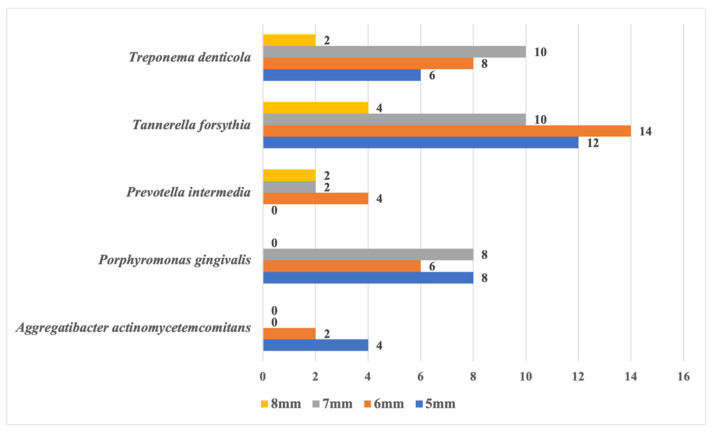
Values of probing depth and positive samples numbers with certain periodontopathogens present.

Regarding the microbiological determinations, as shown in Figure 1, the highest number of periodontopathogenic bacteria was observed in pockets of 5–7 mm, with a few in pockets of 8 mm or more.

*T. forsythia* showed the highest prevalence (20.48%), with decreasing numbers as follow: *T. denticola* (13.31%), *P. gingivalis* (11.26%), *A. actinomycetemcomitans* (7%), and *P. intermedia* (4.9%) (Figure 1).

**Figure 2:**
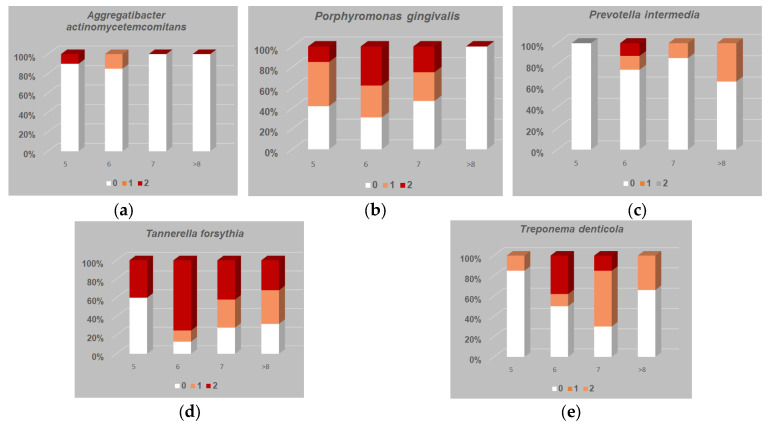
(**a**) *A. actinomycetemcomitans* estimated value in bacterial biofilm samples in periodontal pockets of various probing depths. Probing depth is not significantly correlated to bacterial presence (r = 0.083). (**b**) *P. gingivalis* estimated value in bacterial biofilm samples in periodontal pockets of various probing depths. Probing depth is not significantly correlated to bacterial presence (r = 0.092). (**c**) *P. intermedia* estimated value in bacterial biofilm samples in periodontal pockets of various probing depths. Probing depth is significantly correlated to bacterial presence (r = 0.413, *p* = 0.001). (**d**) *T. forsythia* estimated value in bacterial biofilm samples in periodontal pockets of various probing depths. Probing depth is significantly correlated to bacterial presence (r = 0.305, *p* = 0.002). (**e**) *T. denticola* estimated value in bacterial biofilm samples in periodontal pockets of various probing depths. Probing depth is significantly correlated to bacterial presence (r = 0.275, *p* = 0.018). 0 = not detected; 1 = a weak band [ere + (10^3^) equivalents]; 2 = a clear band [ere + (10^4^)].

As shown in Figure 2, we analyzed the equivalent percentages of bacterial genome in 76 subgingival biofilm units for the following microbes: *P. gingivalis*, *A. actinomycetemcomitans*, *P. intermedia*, *T. denticola*, and *T. forsythia*.

As shown in Figure 2a, regarding *A. actinomycetemcomitans* in 5 mm pockets, a clear band was visible in 10% of cases, whereas at 6 mm probing depths, in 15% of the cases, a weak band was present. Regarding probing depths greater than 7–8 mm, we did not identify any cases with *A. actinomycetemcomitans*.

As shown in Figure 2b, regarding *P. gingivalis*, at a 5 mm probing depth, a clear band was present in 15% of the cases, whereas it was present at 6 mm in 38% of the cases. At a 7 mm pocket depth, in 25% of the cases, a clear band was visible and in 28% of the cases, a weak band was visible. The pockets deeper than 8 mm were negative for *P. gingivalis*.

In Figure 2c, the *P. intermedia* percentages in the samples of biofilm are illustrated. At a 5 mm probing depth, the samples were negative for *P. intermedia*; in the 6 mm pockets in 12% of the cases, a clear band was visible, and in 13% of the cases, a weak band was visible. At a 7 mm probing depth, in 14% of the samples, a weak band was visible; finally, at 8 mm and deeper, in 36% of the instances, a weak band was observed.

As shown in Figure 2d, regarding *T. forsythia* at 5 mm probing depths, in 25% of the instances, a weak band was observed; in the 6 mm periodontal pockets, in 75% of the cases, a clear band was visible, and in 12% of the cases, a weak band was determined. For the probing depths of 7 mm, in 42% of the samples, a clear band was observed and in 30% of the samples, a weak band was observed. Finally, in the periodontal pockets of 8 mm or more, in 32% of the cases, a clear band was present and in 36% of the cases, a weak band was present.

As shown in Figure 2e, regarding *T. denticola* at 5 mm probing depths, in 15% of the cases, a weak band was determined; in the 6 mm periodontal pockets, a clear band was illustrated in 38% of the cases and in 12% of the cases, a weak band was observed. At 7 mm, in 15% of the samples, a clear band was observed and in 55% of the cases, a weak band was observed. In the pockets of 8 mm and beyond, in 34% of the samples, we noted a weak band.

The HbA1c levels showed different values of metabolic control, with an observed range of 5.9–9.6%, and with an average value of 7.1 ± 0.3%.

When evaluating the biofilm samples for the analyzed periodontopathogens in relation to the metabolic control, we observed that *T. denticola* and *T. forsythia* were significantly more commonly observed in individuals with elevated HbA1c serum levels (Table 4).

The *T. forsythia* value for the coefficient of correlation is 0.21 (*p* = 0.01), and 0.44 for *T. denticola*, respectively (*p* = 0.02).

We could not observe any correlation between *P. gingivalis*, *A. actinomycetemcomitans*, and *P. intermedia* presences and the HbA1c value.

## 4. Discussion

The present investigation was conducted on individuals with type I diabetes mellitus and aimed to evaluate certain periodontopathogenic agents in subgingival biofilm samples from periodontal pockets of various probing depths, as well as to compare the values to glycemic control levels, via HbA1c. Our study’s hypothesis was that the pathogenic flora vary in quality depending on the probing depth, and these variations are also associated with different levels of glycemic control.

Both periodontal disease and diabetes are characterized by significant inflammatory phenomena. A series of data from the literature support the influence of periodontopathogenic agents on the metabolic control of diabetes mellitus [27]. The NHANES 2009–2014 analysis observed that a lowered prevalence of periodontitis was associated with factors such as female gender, higher education, and annual dental visits. Meanwhile, socio-economic status, income, smoking, older age (age  >  50 years), and non-white race, were significantly associated with periodontitis prevalence; thus, uncontrolled diabetes was significantly associated with higher odds of periodontal damage among adults in the US [28]. Furthermore, microbiome studies show a potential link between impaired glucose metabolism in diabetes and prediabetes and modifications in the oral ecology. Thus, there is an elevated expression of pathogen receptors in hyperglycemia that upregulate host responses to the altered microbiome [29,30].

The results of our investigation show that the microbiological profile in subgingival biofilm samples differs according to the metabolic control and to the pocket probing depth of diabetic subjects. In our study, we did not observe any periodontal pathogenic agents from the investigated microbial group in the sampled subgingival biofilm from pocket depths equal to or less than 4 mm. The largest percentage of periodontopathogenic genomic equivalents was identified in periodontal pockets of 6 mm and 7 mm depth, with the values illustrating high inflammatory activity. In periodontal pockets the oxygen pressure was low and this environment stimulated the growth of strictly anaerobic or facultative microorganisms and this led to the exacerbation of inflammation and/or infections. In general, these microorganisms do not invade the periodontal tissues on account of the fact that the oxygen pressure in the periodontal tissues is higher than in the periodontal pocket [31].

In subgingival plaques sampled from deep pockets, *T. denticola* and *T. forsythia* were observed most frequently, pathogens that, at the same time, correlated with poorer metabolic control. An area of investigation has focused on the idea that periodontitis can influence the incidence of diabetes mellitus. Ide et al. (2011) conducted a prospective study over a period of 7 years on a cohort of 5848 subjects without a diagnosis of diabetes mellitus. The authors observed that moderate periodontitis (pockets of 3.5–5.5 mm) and severe periodontitis (pockets greater than 5.5 mm) were significantly correlated to an elevated incidence risk of diabetes mellitus; however, the levels of significance after adjustment for factors such as gender, smoking, BMI, hypertension, or lipid profile were lost [32].

Data from our study confirmed previous studies in which the relationship between periodontal status and metabolic control was assessed. We observed a significant association between deep periodontal pockets and affected glucose tolerance, and diabetes, versus shallow pockets (<1.3 mm). In patients with diabetes, we identified *P. gingivalis* more frequently than in subjects without this disease; furthermore, it was most often present in shallow pockets versus deeper pockets. This aspect could be due to the reduced number of deep pockets (7–8 mm) when compared to more shallow–medium depth pockets in our analyzed group; further studies that include a larger number of deep pockets are necessary to elucidate this aspect. Demmer et al. (2010) investigated, in a prospective study over 5 years on 2973 non-diabetic individuals, the impact of periodontal impairment on HbA1c changes. The subjects presenting the most severe forms of periodontitis at the initial moment demonstrated greater absolute elevations in their HbA1c of circa five times over during the 5-year research period, versus the individuals negative for periodontal disease at the initial moment [33].

It is clear that periodontitis is characterized by a modification in oral ecology, with a shift from anaerobic Gram-positive microorganisms (e.g., *Peptostreptococcus*, *Lactobacillus*, *Streptococcus*) to mostly anaerobic Gram-negative microorganisms. The bacterial species implicated in the onset and progression of periodontitis include, among others, *P. gingivalis*, *A. actinomycetemcomitans*, *T. denticola*, and *T. forsythia* [34]. Following the dysbiosis occurring in the subgingival area, the host organism responds to this by developing an inflammatory response, releasing pro- and anti-inflammatory cytokines [35]. This response elaborates on a series of modifiable and non-modifiable factors that can lead to the immune system being unable to resolve inflammation. This inflammation becomes chronic, leading to the generation of ROS, which activate matrix metalloproteinases as a response [36]. Matrix metalloproteinases degrade fibers, such as collagen of the periodontal ligament, resulting in decreased periodontal attachment and increased gingival sulcus depth [37,38].

Periodontitis, through its etiopathogenic mechanisms, has the capacity to increase insulin resistance and disrupt glycemic control; in this sense, Anil et al. (2021) noted elevated levels of periodontopathogens in individuals that have subpar metabolic control [39]. Additionally, the risk of onset and the exacerbated evolution of inflammation in periodontal tissues may be elevated in subjects with decompensated diabetes than in patients with good metabolic control [40,41]. Studies have shown the importance of controlling periodontal disease on improving glycemic control, highlighted by a decrease in insulin demand and a decrease in HbA1c levels [42,43,44].

Due to the fact that periodontal diseases are of microbial etiology, the studies focused initially on the variations in the subgingival microbial flora of diabetic and non-diabetic subjects. Even though some studies have found elevated proportions of particular microorganisms in the subgingival sulcus in individuals with diabetes mellitus, later research that involved cultures have shown minimal differences between diseased periodontal sites in diabetic individuals versus non-diabetics [45]. The majority of studies focused on potential variations in the immuno-inflammatory reaction in individuals with diabetes mellitus and those without [46].

The functions of cells implicated in immuno-inflammatory reply, made up of macrophages, PMNs and monocytes, are modified in diabetic subjects. These cells constitute the host’s first line of defense and the elements of nonspecific defense; dampening their activity can hamper the elimination of microorganisms in the periodontal sulcus, subsequently elevating periodontal loss [47]. Studies found in the literature support the statement that patients with periodontal involvement, in whom the presence of Gram-negative bacteria (*T. forsythia*, *P. gingivalis*, or *P. intermedia*) has been detected, present higher serum values of proinflammatory molecules like fibrinogen, IL-6, or C-reactive protein (CRP) compared to subjects with periodontal health. Moreover, these patients also demonstrated increased insulin resistance, decreasing their metabolic control capacity [48,49,50,51]. Patients with periodontal inflammatory disease frequently present increased levels of proinflammatory cytokines in serum; additionally, in diabetes mellitus individuals, hyperinflammation can increase the generation of proinflammatory cytokines even more. It is possible that these have the potential to elevate insulin resistance and make diabetes control much more difficult for the patient [41].

An interesting finding of our study was offered by the correlation of *T. denticola* and *T. forsythia* with affected metabolic control. These results are somewhat discordant with the literature data, which, for the most part, incriminate *P. gingivalis* for a negative influence. In some previous investigations, *T. forsythia* has been correlated with periodontal destruction, but not with HbA1c value. Aimetti demonstrated a predominance of *T. forsythia* in 21 chronic periodontitis subjects; in 72.22% of the periodontal pockets, *T. forsythia* was identified, whereas *P. gingivalis* was identified in 61.11%, *P. intermedia* was identified in 55.56%, *T. denticola* was identified in 50%, and *A. actinomycetemcomitans* was identified in 33.33% [52].

Regarding the pathogenic potential of *T. forsythia*, it was found that microbial DNA and lipopolysaccharides (LPS) from this microbe cause human macrophages to secrete proinflammatory cytokines; the secretion level of IL-8 of LPS from this bacteria was approximately 1.5-fold higher than the effect of *P. gingivalis* lipopolysaccharides [53,54]. Macrophage cells respond in a complex manner to the presence of *T. forsythia* through autocrine replies to the generated cytokines. According to one hypothesis, the synthesis and secretion of cytokines mediated by periodontal infection can heighten the amount of advanced glycation end products (AGEs) [55]. Moreover, an animal study demonstrated that mice infected with *T. forsythia* exhibited alveolar bone resorption and significant elevated serum amyloid A, lipoproteins, and IgM and IgG antibody levels, and reduced serum nitric oxide versus the controls indicating an altered cholesterol metabolism [56].

Large-scale studies have shown that effective glycemic control can avert or postpone complications’ onset, regardless of the disease duration [57]. Evaluating the level of metabolic control in diabetes is mainly based on monitoring the level of glucose in the blood. Early, occasional, or daily blood glucose determinations have only been carried out to control the glucose level. These measurements still form the basis of daily diabetes management. In a model in which periodontopathogens can stimulate an increased proinflammatory cytokines production, this elevation will favor poor metabolic control, with a hyperglycemic status. Interestingly, periodontal therapy has the ability to reverse this process [58,59].

A study that analyzed the saliva of diabetic and non-diabetic patients indicated that other than well-known periodontitis-inducing pathogens, the proportion of *Ralstonia pickettii*, *Alloprevotella rava*, and *Prevotella copri*, were also significantly elevated in subjects with periodontal disease with or without diabetes. The number of oral pathogens significantly decreased in periodontitis patients with diabetes after effective glycemic control; thus, the researchers concluded that hypoglycemic therapy could potentially rehabilitate the salivary ecology and thus ameliorate the localized oral environment of individuals with periodontitis and diabetes [60].

In individuals with type 1 diabetes, a normal dose of insulin may be insufficient to maintain good glycemic control in the presence of tissue resistance induced by the infectious agent. Chronic infections with Gram-negative bacteria may lead to increased insulin resistance [61]. In patients with periodontal involvement, a persistent challenge from periodontopathogenic bacteria and their products can act similarly to well-known systemic infections. Thus, the aggravation of glycemic control associated with severe periodontitis could be explained through this mechanism. The treatment of periodontal disease aimed at reducing inflammation may lead to the restoration of insulin sensitivity over time, resulting in improved metabolic control [32,62,63,64,65].

Regarding the influence that periodontopathogenic bacteria can have on diabetic patients, numerous mechanisms have been proposed that *T. forsythia* generates effects not only at the periodontal level. This microorganism can influence metabolic control as well as body mass index (BMI), directly affecting the hyperglycemic status. Certain inflammatory molecules can induce cell resistance to insulin effects, exacerbating diabetic status and increasing the risk of complications [66,67,68,69].

High proportions of *T. forsythia* have been the result of significant increases in the depth and prevalence of periodontal pockets in overweight subjects. Other bacteria belonging to the red complex, such as *T. denticola* and *P. gingivalis*, but also including other tested microbes, have not demonstrated this relationship [70]. Furthermore, the presence of *T. forsythia* has been correlated with increased BMI, as well as with a high incidence of gestational diabetes [71].

The pathogenic potential of *T. forsythia* and its influence regarding periodontitis and diabetes was proposed in previous studies and has been corroborated through our studies’ results, proving once more the impact of these bacteria on metabolic control.

Given the presented data, our initial hypothesis has been confirmed. It is proven that diabetes mellitus is linked to a significant risk of augmenting periodontal lesions, and good glycemic control (via HbA1c) can represent a determining factor in this relationship. Research demonstrates numerous plausible biological mechanisms through which these interactions occur, but further investigations are necessary to provide a clear picture of the influence that periodontal pathogens can have on diabetic patients.

The limitations of our study include the reduced number of patients, the use of PCR, the lack of a more comprehensive microbiological analysis such as a microbiome study, reduced follow-up time, the lack of a control group, and limited paraclinical exams.

Treatment strategies for diabetes are needed to address this increasingly important public health issue. Current medical treatments for hyperglycemia include insulin administration and oral antidiabetic drugs. These treatments act to increase insulin sensitivity or availability and thus indirectly reduce hyperglycemia. As a result of improved long-term glucose utilization, a decrease in HbA1c levels is observed.

In the future, potential areas of study could address these issues further, potentially comparing the oral ecology of type I diabetes to type II, including more complete microbiological studies, correlated to systemic status and inflammatory markers and oxidative stress markers. Another possible direction could be how different periodontal therapies affect diabetic status, both in prediabetics and in diabetic patients (controlled and uncontrolled), with an emphasis on surgical and non-surgical therapy and even adjunctive treatments.

## 5. Conclusions

In our study *T. denticola* and *T. forsythia* were most commonly encountered in the sampled subgingival biofilm. Furthermore, the appearance of these periodontopathogenic agents was linked to higher values of HbA1c in patients with type 1 diabetes mellitus. No significant correlation was observed between HbA1c and the presence of *P. gingivalis*, *A. actinomycetemcomitans*, and *P. intermedia*.

In shallow pockets (up to 4 mm), periodontal pathogenic agents from the investigated microbial group were not identified. In the pockets of 6 to 7 mm depth, the greatest percentage of genomic equivalents of periodontopathogenic agents were observed.

## Figures and Tables

**Table 1:** Inclusion and exclusion criteria.

Inclusion Criteria	Exclusion Criteria
Type 1 diabetes patients	Systemic diseases not related to diabetes
Age 18–70 years	History of cancer
Permanent residency in the study area	Pregnancy, lactation
Diagnosed for diabetes type 1 at least 1 year previously	Menopause
	Smokers
	Treatment for periodontal disease in the last 6 months

**Table 2:** Primer and probe sequences.

Pathogen	Primer 5′→3′	Probe 5′→3′	Gene
A. a	F: GCGAACGTTAGCGTTTTAC	AATGCCCGCACCGAAACCCAAC 5′_Cy5→BHQ2_3′	waaA
P. g	F: TGGTTTCATGCAGCTTCTT	GTACCTCATATCCCGAGGGGCTG 5′_HEX→BHQ1_3′	waaA
T. d	F: CCTTGAACAAAAACCGGAA	GAGCTCTGAATAATTTTGATGCA 5′_Cy5→BHQ2_3′	waaG
T. f	F: CTCGCTCGGTGAGTTTGAA	CGATTCGCAAGCGTTATCCCGACT 5′_HEX→BHQ1_3′	waaA
P. i	F: GACCCGAACGCAAAATACAT	CGATTCGCAAGCGTTATCCCGACT 5′_FAM→BHQ1_3′	waaA

Pathogen: P. g = *Porphyromonas gingivalis*; P. i = *Prevotella intermedia*; A. a = *Aggregatibacter actinomycetemcomitans*; T. f = *Tannerella forsythia*; T. d = *Treponema denticola*.

**Table 3:** Demographic data.

Parameter			*p*-Value
Sex	M (n; %)	18; 42.86	0.354
	F (n; %)	24; 57.14	
Age (years)		46.89 ± 1.4	
Residence	Urban (nr; %)	27; 64.29%	0.064
	Rural (nr; %)	15; 35.71%	
HbA1c (%)		7.1 ± 0.3% (range: 5.9–9.6%)	
PD (mm)	4 mm (n)	6	
	5 mm (n)	40	
	6 mm (n)	16	
	7 mm (n)	14	
	>8 mm (n)	6	

HbA1c = Glycated hemoglobin; PD = probing pocket depth.

**Table 4:** Periodontal pathogenic bacteria and association with HbA1c value.

Pathogen		Nr. of Patients	HbA1c (%)	Statistical Analysis
*Tannerella forsythia*	Present	18	7.8 ± 1.9	F-test, *p* = 0.001 r = 0.21, *p* = 0.012
	Absent	24	6.1 ± 0.2	
*Treponema denticola*	Present	16	7.9 ± 1.5	F-test, *p* = 0.001 r = 0.44, *p* = 0.023
	Absent	26	6.4 ± 0.3	

HbA1c: glycated hemoglobin (expressed as mean ± standard deviation).

## Data Availability

The data supporting the reported results can be obtained from the corresponding author upon reasonable request.
